# Fabrication of Effective Nanohybrids Based on Organic Species, Polyvinyl Alcohol and Carbon Nanotubes in Addition to Nanolayers for Removing Heavy Metals from Water under Severe Conditions

**DOI:** 10.3390/molecules27165054

**Published:** 2022-08-09

**Authors:** Hasna Abdullah Alali, Osama Saber, Aya Osama, Mohamed Farouk Ezzeldin

**Affiliations:** 1 Department of Physics, College of Science, King Faisal University, Al-Ahsa 31982, Saudi Arabia; 2 Egyptian Petroleum Research Institute, Nasr City, Cairo 11765, Egypt

**Keywords:** fabrication of nanohybrids, industrial water purification, effective adsorbents in acidic conditions, heavy metal removal

## Abstract

Industrial water has a dual problem because of its strong acidic characteristics and the presence of heavy metals. Removing heavy metals from water in these severe conditions has special requirements. For this problem, an economic method was used for removing iron (Fe), copper (Cu), chromium (Cr), nickel (Ni) and manganese (Mn) with extremely acidic characteristics from water. This method depends on the preparation of nanohybrids through host–guest interactions based on nanolayered structures, organic species (stearic acid), polyvinyl alcohol (PVA) and carbon nanotubes (CNTs). The formation of nanohybrids was confirmed using different techniques through the expansion of the interlayered spacing of the nanolayered structure from 0.76 nm to 1.60 nm, 1.40 nm and 1.06 nm. This nano-spacing is suitable for trapping and confining the different kinds of heavy metal. The experimental results indicated that the prepared nanohybrid was more effective than GreensandPlus, which is used on the market for purifying water. The high activity of the nanohybrid is obvious in the removal of both copper and nickel because the GreensandPlus was completely inactive for these heavy metals under severe conditions. Finally, these experimental results introduce new promising materials for purifying industrial water that can work under severe conditions.

## 1. Introduction

There is no doubt that pollutants, heavy metals, salts, and poisons can damage bone, liver and kidney, cause gastrointestinal distress, damage nerves in the human body and deactivate the functional groups of important enzymes [1,2,3]. According to the Environmental Protection Agency (EPA), the limit values for iron (Fe), copper (Cu), chromium (Cr), nickel (Ni) and manganese (Mn) ions in drinking water are 0.3, 1.0, 0.1, 0.1 and 0.05 mg/L, respectively [3,4]. Hence, to protect human health, groundwater sources and other water bodies that receive contamination from industrial discharge must be treated.

Many studies have reported the purification of groundwater sources [1,2,3]. However, there are not enough studies on the treatment of industrial water. However, industrial wastewater is not only a by-product of oil and gas companies, mining and chemical manufacturing companies; it is also a by-product of the food- and beverage- processing industries, which are necessary for the production of clothes, the shoes on your feet, the computer at your fingertips, and the car you drive. For example, in the metal-finishing industry, the wastewater is often a slurry comprising metals that have been dissolved in liquid. Iron, manganese, nickel, zinc, copper and chromium are generated during metal plating, metal finishing, and printed circuit board (PCB) manufacturing activities. In the manufacturing of iron and steel, water is used for cooling and by-product separation. At the same time, hydrochloric acid and sulfuric acid are required in the water used for galvanizing steel. Wastewater contains acidic-rinse waters as well as waste acids. Shale gas drilling wastewater is classified as hazardous waste. It is extremely salty. Furthermore, water containing high quantities of sodium, magnesium, iron and manganese is put into the well to ease digging. Therefore, the current study focused on designing strong and stable materials to work in a strong acidic medium for the removal of the most familiar heavy metals that exist in industrial water.

Generally, several water and wastewater treatment techniques have been established [1] (e.g., precipitation/coagulation, electro-dialysis, ion exchange and adsorption). Even for treated wastewater used for agriculture purposes, the Saudi Ministry of Municipal, Rural Affairs and Housing declared maximum limit values for the occurrence of heavy metals in reused tertiary treated wastewater for agriculture purposes. For example, the limit concentrations of Fe, Cu, Cr, Ni and Mn are 5.0, 0.4, 0.1, 0.2 and 0.2 mg/L, respectively [5]. In fact, several approaches have been stablished to choose the specific water and wastewater treatment technique depending on the concentration of cations/anions, the water source, the eco-friendliness and the cost of treatment [1]. Nowadays, wide applications of nanomaterials in different fields have been recognized and reported by many scientists as shown in Table 1.

Additionally, one more criterion is well known, which is the high mobility of nanomaterials in solution [11]. Indeed, the application of nanomaterials in water and wastewater treatment is one of the most rapidly advancing fields of science. Different forms of nanostructures have been generated for the purpose of water purification such as nanolayered structures, nanohybrids, carbon nanotubes and nanocomposites [12]. Many studies about the removal or partial removal of heavy metals [13], organic pollutants [14], inorganic anions [15,16] and bacteria deactivation [17] using nanomaterials have been published.

Layered double hydroxides (LDHs) can be defined as anionic clay, and are basically a type of hydrotalcite-like compound. Various advantages of LDHs have been described such as high resistance to the changing pH and temperature of the medium, high surface area, memory effect properties, and a high capacity for anion exchange treatments, as well as a low cost of production [18]. However, despite all these great features, the practical applications of pure LDHs are limited. A large charge density as well as a high hydrophilic nature are known disadvantages of using LDHs in significant industrial applications. Their low tendency to interact with the compounds of hydrophobic linkages and their intramolecular structure with high aggregation properties are the main reasons for this restriction [19]. Consequently, the need to develop pure LDH structures to overcome these weaknesses can be achieved through the interaction with adjusting agents and/or the consideration of new preparation methods. Actually, both organic and inorganic modifiers may be used to treat the surface and interlayer space of LDHs for modification purposes [20].

Generally speaking, the manufacture of LDHs usually depends on the usage of an extended range of divalent and/or trivalent cations accompanied by various interlayer anions [19,21]. However, many factors should be considered when the synthesis of such an LDH structure is required. For example, the method of preparation, the characteristics of both the interlayer anions as well as the metallic cation, the morphological properties and crystalline structure are the most important aspects [22]. Consequently, the produced LDH material gains different physical characters, and this leads to distinct industrial applications. Many fabrication methods have been issued for the production of several kinds of LDHs. The most common methods can be considered to be the co-precipitation, anion-exchange, hydrothermal, urea-hydrolysis, microwave, and sol-gel methods [23,24,25,26,27].

Recently, many studies have been published in the field of the remediation of different pollutants from water and wastewater by developing LDH structures. These reports were devoted to eliminating organic [28,29] and inorganic pollutants in water [30,31,32]. This demonstrates the fast development of nanomaterials, in general, and of the LDH format, in particular; however, further research is needed to improve the synthesis as well as final characteristics of the developed LDHs for them to be active and stable for the purification of water under severe conditions.

Various hetero-structured hybrids, such as organic–inorganic [23,26] systems, have attracted considerable research interest due to their unusual physicochemical properties which cannot be achieved via conventional solid-state reactions. This field appears to be very creative, since it gives rise to an almost unlimited set of new compounds (hybrid compounds) with a large spectrum of known and unknown properties. The nanolayered structures of LDHs can be used as hosts for organic guests that require functional groups to be compatible with the polymers.

Therefore, the current study used stearic acid as a guest for LDH, which acted as a host to form an organic–inorganic nanohybrid material and become compatible with the other additives of polyvinyl alcohol and carbon nanotubes. Hence, the current study aims to address new modified LDH material(s) able to significantly remove more than one heavy metal from water under severe conditions. In this way, a series of modifications were used for developing the nanolayered structures of LDHs. Organic species were intercalated inside the LDHs to control the interlayered spacing of the nanolayered structures through the formation of organic–inorganic nanohybrids. In addition, carbon nanotubes and polyvinyl alcohol were used as fillers for the LDHs to increase the stability of the nanolayered structures of the LDHs. Through these modifications, a series of nanolayered structures and nanohybrids were prepared and used for purifying water containing different kinds of heavy metals under severe conditions.

## 2. Results

### 2.1. Characterization and Effect of Nanolayered Structures on Heavy Metal Removal

**Figure 1:**
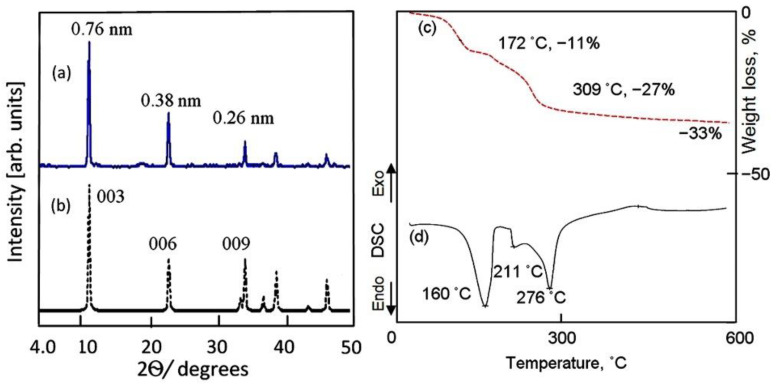
Sample AZ-1: (**a**) X-ray diffraction, (**b**) standard JCPDS file No. 48-1022, (**c**) TG curve and (**d**) DSC curve.

X-ray diffraction (XRD) was used to identify the layered structure of the prepared Al/Zn LDHs. An XRD diagram shows the characteristic peaks of the layered structure of the natural hydrotalcite for sample AZ-1, which was composed of zinc and aluminum, agreeing with standard JCPDS file No. 37-629. Additionally, it matches with standard JCPDS file No. 48-1022 of the synthetic Zn–Al LDH, as shown in Figure 1a,b.

A sharp peak is observed for the main plane (003) at d = 0.76 nm, as shown in Figure 1b. At the same time, the XRD diagram exhibits other peaks for planes 006 and 009 at d = 0.38 nm and d = 0.26 nm, respectively. In addition, Figure 1a,b indicates that there is a clear arrangement between these values of the basal planes, as seen in the following: d_(003)_ = 2 × d_(006)_ = 3 × d_(009)_. This means that the nanolayered structure of AZ-1 has highly ordered nanolayers along axis “c”. The value “c” represents the thickness of the brucite-like layer and the interlayer distance. Depending on the interlayered spacing of plane (003), the value “c” was assessed by 3 × d_(003)_ to equal 2.28 nm. It is completely matched with the value “c” of hydrotalcite, with a value of 2.28 nm [14,15].

Thermal measurements (thermogravimetric analysis (TGA) and differential scanning calorimetry (DSC)) were taken to confirm the nanolayered structure formation and identify the thermal behavior of the internal contents of the AZ-1 sample. The recorded thermogravimetric and differential scanning calorimetric diagrams are shown in Figure 1c,d. The thermal degradation of the internal contents of AZ-1 was observed at several stages with various mass-loss rates, depending upon the nature of the interlayer anions. As can be seen from the TG curves of AZ-1 in Figure 1c, the degradation process exhibited four weight losses. The first mass loss (11 wt.%) was detected at a temperature of 172 °C, which can be attributed to the loss of both the physisorbed and interlayer water. The other three mass losses (22 wt.%) were observed at higher temperatures (172–600 °C). These transitions can be attributed to the decomposition of the interlayered anions in addition to the dehydroxylation process. It means that the nanolayered structures of AZ-1 have two different anions with two different mass-loss rates. This finding is confirmed a DSC curve. The DSC curve of AZ-1 shows three endothermic peaks at 160 °C, 211 °C and 276 °C; this agrees with the removal of the intercalated water and the decomposition of the intercalated cyanate anions in addition to the degradation of the intercalated carbonate anions, as shown in Figure 1d [14,15].

**Figure 2:**
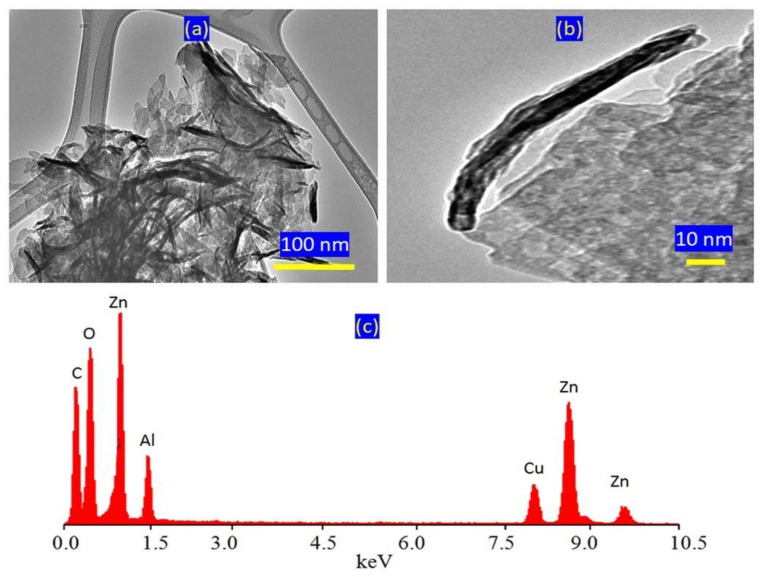
Sample AZ-1: (**a**) TEM image at 100 nm, (**b**) TEM image at 10 nm and (**c**) EDX spectrum.

The previous studies showed that natural samples of layered double hydroxides (pyroaurite) have a plate-like morphology. In addition, the dimensions of these plates are in millimeters and not at the nano scale [31]. It is known that the morphology of hydrotalcite, which is carefully crystallized, is a hexagonal platy [33]. A similar morphology is observed for AZ-1, with the dimensions of the plates in the order of 100 nm, as shown in Figure 2a. Additionally, Figure 2a indicates strong aggregates of nanoplates. Via magnification, it was shown that the thickness of the individual plate was 10 nm (Figure 2b). The of energy-dispersive X-ray spectrometry analysis revealed that inorganic elements (zinc and aluminum) were clearly identified in the platelets of sample AZ-1 as shown in Figure 2c. Additionally, interlayered anions were confirmed by signals of carbon and oxygen at low energy. It should be mentioned that the Cu signal, which was observed in the spectrum, is attributed to the copper grid of the TEM testing.

In order to determine the ability of the prepared nanolayered structure AZ-1 to purify water containing heavy metals, adsorption experiments were performed for an aqueous standard solution containing iron (Fe), copper (Cu), chromium (Cr), nickel (Ni) and manganese (Mn) with an extremely acidic medium (pH 2~3). The original concentrations of the analyzed standards are reported in Table 2.

**Figure 3:**
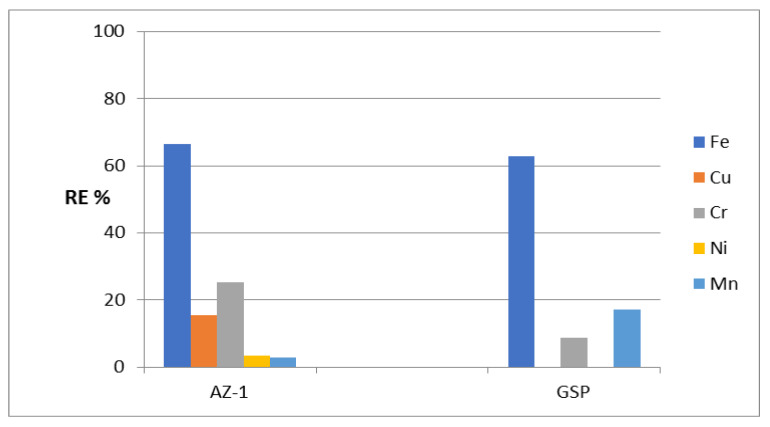
The removal percentage of heavy metals from water in an extremely acidic medium for the AZ-1 samples and the commercial material GreensandPlus (GSP).

By using AZ-1 as an adsorbent (W/V = 2 g/L), high removal of iron was observed. This shows that the RE% of Fe is 66%, as seen in Figure 3. In the case of the removal of chromium and copper, the percentage became lower because the RE% values of Cr and Cu were 25% and 15%. For nickel and manganese, the AZ-1 sample was not effective where the RE% values of Ni and Mn were 4% and 3%, respectively. The high removal of iron by AZ-1 can be explained by its nanolayered structure.

**Figure 4:**
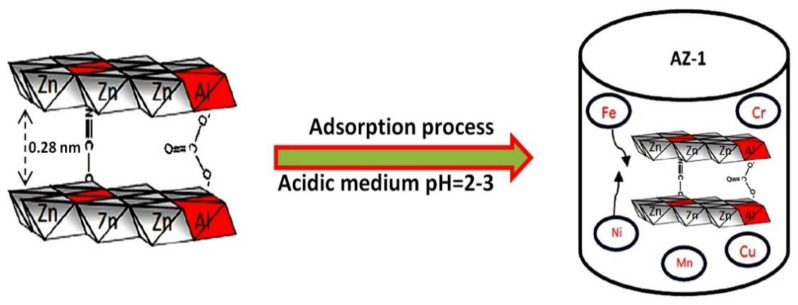
Adsorption process for purifying water containing heavy metals in an extremely acidic medium.

It is known that the size of the nanolayers is 0.48 nm [14,15]. According to the calculations of X-ray diffraction, we can suggest that the interlayered spacing of the nanolayered structures is 0.28 nm, which is suitable and favorable for trapping and confining the iron particles among the nanolayers, as shown in Figure 4.

GreensandPlus (GSP) was used as a model of real commercial material available on the market for comparison. GreensandPlus is well-known as a common filter for the removal of iron, manganese, hydrogen sulfide and arsenic for many commercial and industrial water treatment plants. It is also branded as a black filter, mainly composed of silica sand as a support material, coated with fused manganese dioxide. In fact, water treatment with this saleable media consists of a kind of catalytic oxidation-reduction reaction of Fe and Mn in applied water.

Actually, one can understand that the optimum operation conditions for effective Greensand filtration are the feeding of naturally neutral water or neutralized water—most likely with a pH of 6.7 to 8.8—as well as a total combined concentration of both Fe and Mn of preferably less than 15 PPM in feed water. The current study took place under severe water treatment conditions. However, the pH was extremely acidic (pH 2~3) alongside numerous concentrations of many heavy metals which amounted to about 90 PPM (a total combined concentration of Fe and Mn of about 40 PPM) as shown in Table 1; the prepared tested materials and GreensandPlus were still able to resist these conditions without breakdown. The RE% values achieved using “GreensandPlus” were 62%, 18% and 9% for Fe, Mn and Cr, respectively, whereas no adsorption was noticed towards Cu and Ni. Compared with the results of GreensandPlus, the prepared sample AZ-1 is more favorable and useful than GreensandPlus for removing iron and chromium.

### 2.2. Characterization and Effect of CNT–PVA Inorganic Nanohybrids on Heavy Metal Removal

Many efforts by researchers have created an interest in developing nanostructures through combination with carbon nanotubes. Therefore, functional carbon nanotubes were used as additives during the building of the Al/Zn nanolayered structures to produce the AZ-2 sample. At the same time, polyvinyl alcohol was used as a binder for the nanolayered structure.

**Figure 5:**
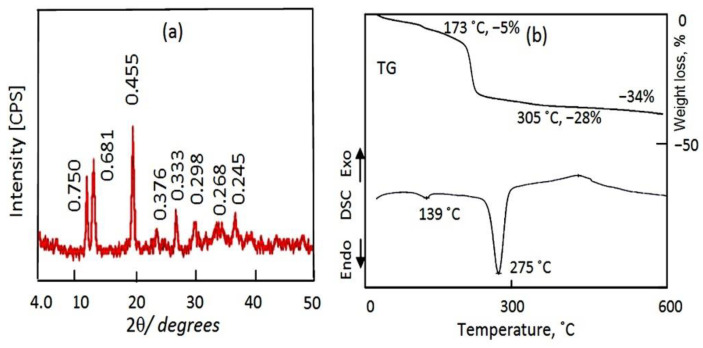
The AZ-2 sample: (**a**) X-ray diffraction, and (**b**) thermal analyses.

The AZ-2 sample was characterized using different techniques to identify the texture of its nanolayered structures. Figure 5a revealed the X-ray diffraction pattern. It showed that the AZ-2 sample consists of two nanolayered structures.

The first structure is made clear by observing three peaks at 2Ѳ = 11.79°, 23.40° and 33.35°, which agree with the d-spacing of 0.750 nm, 0.376 nm and 0.268 nm. The good arrangement and similarities between the value of 0.750 nm, the double value of 0.376 and the triple value of 0.268 nm indicate a nanolayered structure, agreeing with the hydrotalcite-like materials (JCPDS file No. 37-629). This means that the first structure of AZ-2 is the nanolayered structure of zinc aluminum hydroxide carbonate hydrate.

The second nanolayered structure can be observed in the peaks at 2Ѳ = 12.99°, 26.74° and 36.60° which agree with the d-spacing of 0.681 nm, 0.333 nm and 0.245 nm, as shown in Figure 5a. The similarities among the d-spacing of the main peak 0.681 nm and the double value of the second peak (2 × 0.333 nm), in addition to the triple value of the third peak (3 × 0.245 nm), confirms the production of a new nanolayered structure through combination with carbon nanotubes and polyvinyl alcohol. According to the calculations of XRD, the second nanolayered structure represents 50% of the AZ-2 sample.

Additionally, Figure 5a showed a strong and sharp peak at 2Ѳ = 19.50°, agreeing with the d-spacing of 0.445 nm. This peak is characteristic of polyvinyl alcohol and is due to the reflection of plane (101) [5]. It is known that pure polyvinyl alcohol has a semi-crystalline structure [6,7]. The crystalline nature of PVA is due to the hydrogen-bonding of the hydroxyl groups of PVA, which causes small crystallites in the amorphous PVA matrix and is related to PVA’s semi-crystalline structure [8]. The high crystallinity of PVA, which is observed in Figure 5a, means that its combination with the nanolayered structure converted PVA from a semi-crystalline material to a crystalline material by increasing the H-bonds of the chains of PVA with the nanolayers of Al/Zn LDHs and CNTs. Through the XRD results, we concluded that the addition of CNTs to PVA during the preparation of the AZ-2 sample produced two nanolayered structures. The first structure is the normal nanolayered structure of Al/Zn LDHs, while the other one combined with the chains of PVA and carbon nanotubes through hydrogen bonds, which decreased the interlayered spacing of the nanolayered structure from 0.750 nm to 0.681 nm.

Thermal analyses confirmed the nanolayered structures, as shown in the TG curve and DSC diagram in Figure 5b. The TG curve shows that 28% of the sample was lost after heating up to 305 °C, as seen in Figure 5b. This means that 28% of the content of the sample, which consisted of water, PVA and anions, was confined in the interlayered region. In addition, the hydroxyl groups of the nanolayers, which represent 6%, were removed via heating up to 600 °C. The DSC diagram shows two endothermic peaks and one exothermic peak, confirming TG results. The two endothermic peaks are observed at 139 °C and 275 °C, agreeing with the removal of the interlayered water and anions, respectively. Additionally, the exothermic peak is due to crystallization process after the removal of hydroxyl groups.

**Figure 6:**
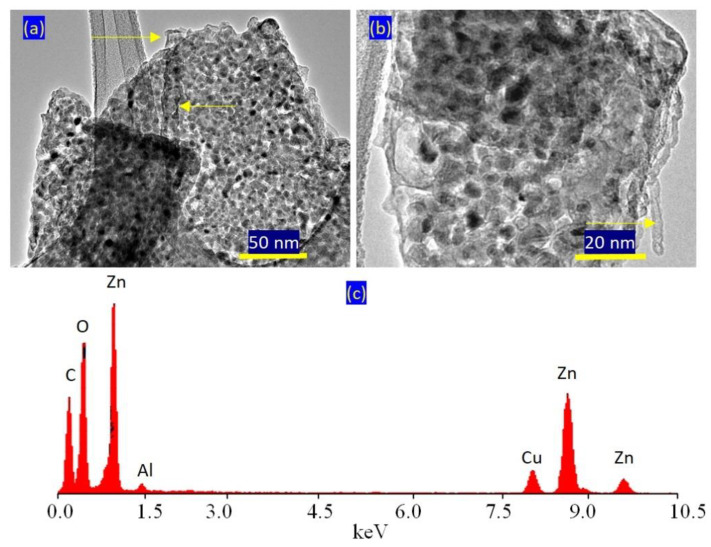
The AZ-2 sample: (**a**) TEM image at 50 nm, (**b**) TEM image at 20 nm and (**c**) EDX spectrum.

TEM images confirmed the presence of two nanolayered structures. Figure 6a shows clear and large plates with irregular shapes for the normal nanolayered structure of Al/Zn LDHs. The second nanolayered structure, which was modified by CNTs and PVA, is shown in Figure 6a and is marked by yellow arrows. In Figure 6b, the CNTs are clear and marked by a yellow arrow. This means that the nanolayered structures grew around the CNTs, agreeing with the XRD results. EDX analysis confirmed the presence of inorganic species of zinc, aluminum and oxygen, as shown in Figure 6c. Additionally, the CNTs were observed as a clear carbon peak at low energy.

**Figure 7:**
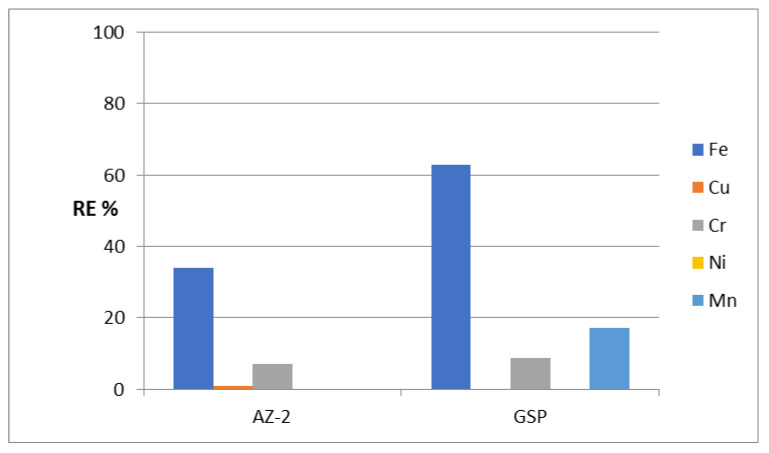
The removal percentage of heavy metals from water in an extremely acidic medium for the AZ-2 and GreensandPlus samples.

After identifying the structure of the AZ-2 sample, its ability to remove heavy metals from water was tested through the same adsorption process. Using the AZ-2 sample resulted in low activity for purifying water containing heavy metals, whereas the RE% of AZ-2 for iron was 33.9% as shown in Figure 7. This low activity of the AZ-2 sample was very clear in the removal of chromium and copper. The removal percentages of both Cr and Cu were 7.14% and 1.06%, respectively. In the removal of nickel and manganese, there was no activity for the ZA-2 sample.

Compared with GreensandPlus, the activity of the AZ-2 sample under severe conditions was lower in the removal of iron and chromium. Additionally, for copper, the activity was very low. In the case of nickel and manganese, the AZ- 2 sample was completely inactive. The low activity of the AZ-2 sample can be explained by the presence of CNTs and PVA. The H-bonding of the PVA chains and CNTs caused narrowing by 50% of the interlayered spacing of the nanolayered structure, from 0.750 nm to 0.681 nm. This means that CNTs and PVA occupied part of the empty space among the nanolayers of the AZ-2 sample. In addition, they caused a blockage of the entrance gate of the interlayered region of the nanolayered structures. These effects led to a weakened ability of the nanolayered structures in the removal of heavy metals from water.

In order to avoid these blockage effects, the entrance gate of the nanolayered structure needs to expand or widen through the intercalation of fatty aliphatic acid with long chains of hydrocarbons to act as long pillars. In the next section, we describe how the nanolayered structure was modified and developed via intercalation reactions to build organic-CNT–PVA inorganic nanohybrids.

### 2.3. Characterization and Effect of Organic-CNT–PVA Inorganic Nanohybrids on Heavy Metal Removal

In order to expand and widen the entrance gate of the nanolayered structures, fatty aliphatic acid with long chains of hydrocarbons were used to increase the interlayered spacing through the building of organic-CNT–PVA inorganic nanohybrids via intercalation reactions. By intercalating stearic acid (CH_3_(CH_2_)_16_COOH) inside the nanolayered structure, the AZ-3 sample was prepared and characterized via X-ray diffraction, thermal analyses and FTIR, in addition to TEM and EDX.

**Figure 8:**
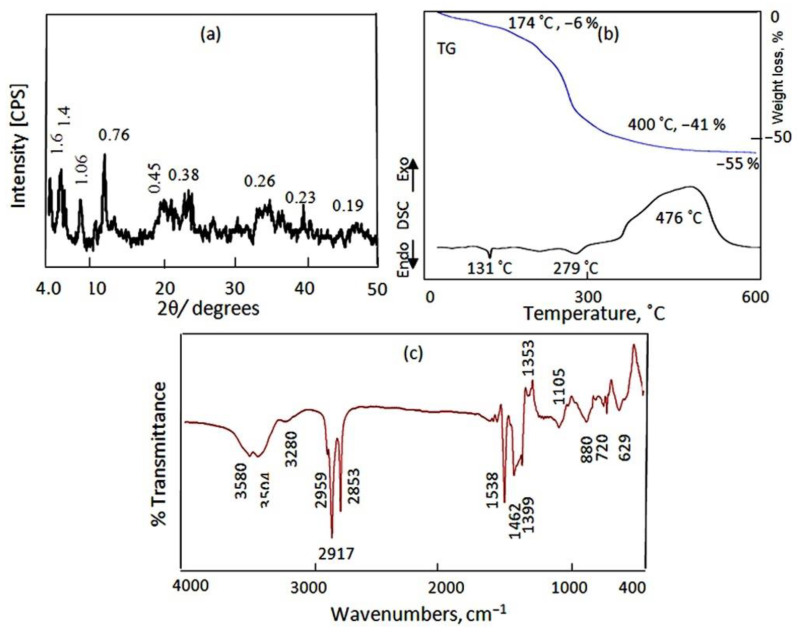
The AZ-3 sample: (**a**) X-ray diffraction, (**b**) thermal analyses and (**c**) FTIR.

Figure 8a revealed the X-ray diffraction pattern of the AZ-3 sample. It showed three clear peaks at low 2Ѳ of 4.1°, 6.2° and 8.3°. These peaks indicate that a new nanolayered structure was formed after the intercalation reactions. In this nanolayered structure, the interlayered spacing increased to 1.6 nm, 1.4 nm and 1.06 nm. Additionally, the normal nanolayered structure of Al/Zn LDHs was observed in the XRD pattern. Figure 8a shows the main characteristic peaks of Al/Zn LDHs at 11.6°, 23.4° and 34.5°, agreeing with the d-spacing of 0.76 nm, 0.38 nm and 0.26 nm. These three peaks are related to the reflections of the (003), (006) and (009) planes, which is in agreement with the standard data for zinc aluminum hydroxide carbonate hydrate (JCPDS no. 48-1022). Additionally, planes (015) and (018) were observed at 0.23 nm and 0.19 nm, confirming the nanolayered structure of Al/Zn LDHs. In addition, Figure 8a shows a broad peak at 0.45 nm, indicating the presence of polyvinyl alcohol inside the nanolayered structure of AZ-3. According to the XRD results, the AZ-3 sample consists of two different nanolayered structures. The first structure is a nanohybrid with interlayered spacing of more than 1.5 nm. The second structure is the normal nanolayered structure with interlayered spacing of 0.76 nm.

The thermal analyses confirmed the formation of a nanohybrid through, as shown in the TG curve and DSC diagram in Figure 8b. The TG curve shows that 55 wt.% was lost via heating up to 600 °C. This means that more than 50% of the structure of the AZ-3 sample was concentrated in the interlayered region as water and anions, confirming the formation of a nanohybrid structure. The DSC diagram confirms the TG data through a broad and a large exothermic peak at 476 °C, indicating oxidation reactions of the hydrocarbon species. Additionally, the DSC diagram shows two small endothermic peaks at 131 °C and 279 °C, indicating the presence of the normal nanolayered structure of Al/Zn LDHs as a secondary phase. These analyses concluded that the AZ-3 sample had two nanolayered structures with two interlayered spacings, one below 1 nm and the other above 1.5 nm.

FTIR confirmed the presence of a nanohybrid through sharp bands at 2959 cm^−1^, 2917 cm^−1^ and 2853 cm^−1^. These bands are due to the C–H stretching absorption bands of organic species. Additionally, a bending band of C–H is observed at 1462 cm^−1^ in Figure 8c. In addition, asymmetric and symmetric stretching vibrations of carboxylate are observed at 1392 cm^−1^ and 1538 cm^−1^, respectively. The hydroxyl groups of the nanolayers of the AZ-3 sample are observed through two bands at 3580 cm^−1^ and 3280 cm^−1^. This means that there are two types of hydroxyl groups, confirming the presence of two nanolayered structures. The first type of hydroxyl group is due to the normal nanolayered structure of Al/Zn LDHs. The second type of hydroxyl group, which is affected by hydrocarbon chains and polyvinyl alcohol, belongs to the nanolayered structure of the Al/Zn nanohybrids.

**Figure 9:**
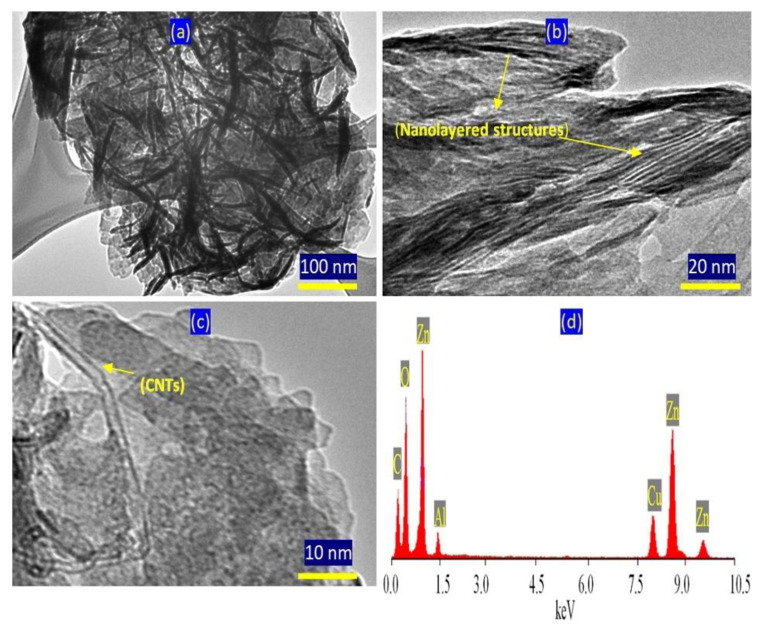
TEM images of AZ-3: (**a**) at 100 nm, (**b**) at 20 nm, (**c**) at 10 nm and (**d**) in the EDX spectrum.

Figure 9 shows the TEM images and EDX spectrum of the AZ-3 sample. A plate-like morphology is observed in Figure 9a. The images of the AZ -3 sample magnified up to 20 nm indicate that the Al/Zn nanohybrids have clear nanolayered structures, as seen in Figure 9b. Higher magnification of the AZ-3 sample revealed intercalated CNT among the nanolayers, as shown in Figure 9c. The EDX analysis confirmed that the nanohybrid structure of AZ-3 consists of zinc, aluminum and oxygen, in addition to carbon, as shown in Figure 9d, wherein the EDX spectrum reveals sharp peaks for zinc and oxygen. At the same time, weak peaks are observed for aluminum and carbon.

**Figure 10:**
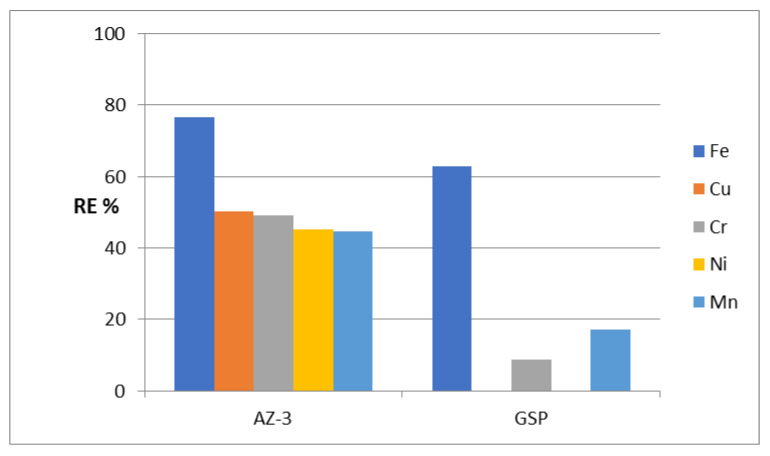
The removal percentage of heavy metals from water in an extremely acidic medium for the AZ-3 and GreensandPlus samples.

To evaluate its ability to remove heavy metals from water under severe conditions, an adsorption process was used for the five metals iron, copper, chromium, nickel and manganese. High activity was observed for the AZ-3 sample in removing iron from water. Figure 10 shows that 76.6% of iron was removed by the AZ-3 sample. This high activity continued for the removal of both copper and chromium because the RE% values of Cu and Cr were 50.2% and 49.1%. Displaying the same trend, Figure 10 shows high removal of nickel and manganese. Removal percentages of 45.1% and 44.6% were observed for nickel and manganese, respectively.

Compared with GreensandPlus, the AZ-3 sample showed higher activity for the removal of heavy metals from water; the removal percentage of iron increased from 61% to 76.6% as a result of replacing the AZ-3 sample instead of the GreensandPlus. This high activity became clear through a comparison of the removal percentage of chromium using GreensandPlus with that using the AZ-3 sample. This comparison indicates that the activity of the AZ-3 sample is three times higher than that of GreensandPlus. For manganese, the removal percentage became more than double after using the AZ-3 sample. Displaying the same trend, Figure 10 shows that the high activity of the AZ-3 sample is obvious in the removal of both copper and nickel because the GreensandPlus was completely inactive for these heavy metals.

This high activity of the AZ-3 sample can be explained by the texture of its nanolayered structure. It has different interlayered spacing: 0.75 nm, 1.06 nm, 1.40 nm and 1.60 nm. At the same time, the heavy metals have different sizes. This can give them a good chance of trapping different kinds of heavy metals. Additionally, the long chains of hydrocarbons widen the entrance gate for interlayered spacing to provide easy penetration and allow heavy metals to enter the nanolayered structures. In addition, the CNTs and PVA can trap and prevent heavy metals from leaving the nanolayered structures. These advantages led to high performance and stability in the removal of heavy metals under severe conditions.

## 3. Discussion

**Figure 11:**
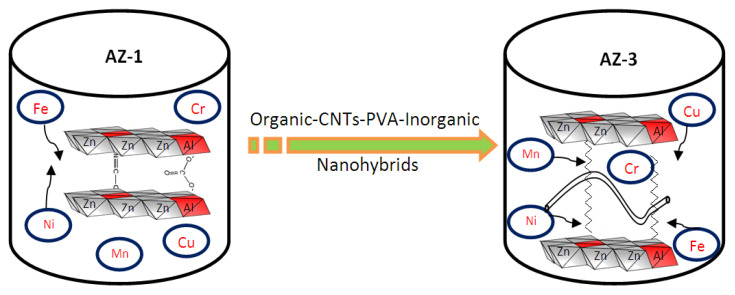
The removal process of heavy metals from water in an extremely acidic medium for the AZ-3 samples.

The current study shows that the modification which was used for developing nanolayered structures in LDHs introduces a new effective material for working under severe conditions to purify water containing different kinds of heavy metals. Organic species were intercalated inside the LDHs to expand the interlayered spacing of the nanolayered structures through the formation of organic CNT–PVA inorganic nanohybrids and through widening of the entrance gate of the nanolayered structures for easy access of the heavy metals. In addition, carbon nanotubes were used as fillers for the LDHs to increase the stability of the nanolayered structures of LDHs, in addition to the trapping and confinement of the heavy metals inside the nanolayered structure. Through these modifications, the nanohybrids, which were modified by the carbon nanotubes and the organic species, were effective and active in purifying water containing five of the heavy metals (Fe, Cu, Cr, Ni and Mn) under severe conditions, as shown in Figure 11.

Figure 11 showed that the long chains of hydrocarbons of stearic acid expanded and widened the interlayered spacing to higher than 1 nm. This spacing allowed the carbon nanotubes to intercalate among the nanolayers of the nanohybrids. In addition, the entrance gates of the nanolayered structures accelerated the insertion of heavy metals among the nanolayers. At the same time, the carbon nanotubes confined the heavy metals inside the nanolayered structures and prevented them from exiting again. This behavior is similar to that of trapping and catching fish through nets.

Wang et al. [31] studied the modified structures of LDH based on a sulfide-selector intercalated, layered, double hydroxide adsorbent for purifying water containing only two metals (Cu and Mn) under normal conditions. Their results indicated that their materials were active for copper (100% removal) and inactive for manganese (0.66% removal). Compared with the results of Wang et al., our product was more effective.

Recently, one interesting research paper about the application of LDHs for the elimination of some heavy metal (Fe, Cu and Cr) ions from the discharge of industrial wastewater was conducted by Cardinale et al. [33]. In this work, two different LDH materials, MgAl-CO_3_ and NiAl-NO_3_, were fabricated to remove, by adsorption, some pollutants from a galvanic plant effluent. Although there was a high concentration of Fe, Cu and Cr ions in the original sample and a low pH value (3), the researchers adapted the experimental conditions to avoid the severe conditions and the decomposition of synthesized materials by diluting the sample and raising the pH to 5. Interestingly, the NiAl-NO_3_ LDH structure showed higher affinity for the Cr (almost 99%) ion than for the Fe and Cu ions (88% and 55%), respectively. In contrast, MgAl-CO_3_ LDH had a lower affinity for adsorbing Cr ion (only 29%), whereas it was very powerful for the other two ions (almost 99%). Comparing the results of Cardinale et al. with the present work, the prepared materials resisted the high acidity of the medium (pH 2~3) and was able to treat Fe, Cu and Cr ions with satisfactory removal efficiencies of 85%, 80% and 70%, respectively. In addition, two more heavy metals were reasonably removed, whereas Ni and Mn ions were adsorbed with a removal efficiency of more than 60%, as were the previously mentioned elements.

Asiabi et al. [34] studied the removal of copper using six samples of Ni-Cr LDHs. Although this study achieved an excellent removal percentage of Cu from aqueous solution as individual ions (almost 100%), this percentage did not exceed 31% during the adsorption of the best LDH structure (diphenylamine-4-sulfonate (DPA) in the surrounding solution of four different mixed ions (Cd, Cu, Zn and Pb). In addition, the adsorption process was achieved under normal conditions (pH = 6).

The removal of Cr ions was conducted by Tan et al. [35]. They concluded that CMC–LDH beads effectively removed high concentrations of Cr ions in aqueous solution which was previously optimized, with a pH in range of 6–7. Although, the adsorption ability of the studied material was powerful, the experimental conditions were adjusted for an individual ideal Cr ion standard solution. In other words, there was no competition regarding the Cr ion studied with other types of heavy metals that may be found in real water or wastewater samples.

A study on an individual metal iron (II) removal from aqueous solution was conducted by Taher et al. [36]. The results of this study proved that the elimination of Fe^2+^ using intercalated Ca/Al LDH with a Keggin ion was higher in adsorption-capacity factor than the original Ca/Al LDH. Additionally, the stability and the highest adsorption capacity of modified structures were diagnosed at low PH, which is very similar to the findings of the current research. The output of another research paper by Oktriyanti et al. [37] confirmed the greater power of a modified structure of LDH regarding the removal of iron from aqueous solution under acidic conditions than pristine LDH.

It was rare to find a study about the removal of nickel ions by via LDH. However, a recent study on the elimination of Ni^2+^ from water via nanoparticle structure (Fe/MOP) was discussed [38]. This study declared the removal of Ni ions only from water over 90% under the designed optimum conditions. These conditions were an alkaline medium (pH ~ 8), a temperature of 37 °C and a contact time of 85 min.

Hence, the present modified structures prepared via CNTs and organic species can be characterized as a multi-ion-selective materials working under non-optimized severe conditions. Consequently, it is very clear that, in reference to the above-mentioned studies, the current modified structures showed a couple of advantages. The first one is the ability to work under severe conditions in a strong acidic medium. The second advantage is the ability to remove many heavy metal (Fe, Cu, Cr, Ni and Mn) ions in the same solution at the same time.

## 4. Materials and Methods

### 4.1. Preparation of Nanolayered Structures and Nanohybrids

Three samples of Al/Zn nanolayered materials were prepared and modified using different techniques. The standard sample was prepared via urea hydrolysis by reacting an aqueous solution (0.07 mol) of zinc nitrate with aluminum nitrate in the presence of urea. The molar ratio between Zn/Al was 3. The reaction was carried out at 90 °C for 12 h to obtain a white precipitate. The sample was filtrated and washed using deionized water several times. After drying at room temperature, the white powder was collected and coded using AZ-1. An amount of 0.1 g of carbon nanotubes (CNTs) and 0.5 g of polyvinyl alcohol (PVA) were used as seeds for building the Al/Zn nanolayered structure. In the presence of CNTs and PVA, the second sample was precipitated after 12 h of reaction at 90 °C. A grey precipitate was formed. This precipitate was washed several times with hot water to remove the excess PVA. The sample was dried and coded using AZ-2. The third sample, AZ-3, was modified by intercalating the organic species, in addition to CNTs and PVA, to build organic-CNT–PVA inorganic nanohybrids through the host–guest technique. Therefore, 1 g of stearic acid sodium salt was dissolved in an aqueous solution of zinc nitrate and aluminum nitrate in the presence of urea. Additionally, PVA and CNTs were used as additives during the precipitation of the nanohybrid, as mentioned in the preparation of the second sample. Urea and polyvinyl alcohol were used as a pH controller and a binder, respectively. The temperature of the reaction in all preparations was kept at 90 °C for 12 h. The products were washed with deionized water. They were dehydrated at room temperature for 48 h.

### 4.2. Physical Characterization

X-ray diffraction (XRD) is considered one of the main tools for determining nanolayered structures. For X-ray diffraction (Bruker-AXS, Karlsruhe, Germany), we used wide-angle X-ray scattering over 2θ = 4 to 50°, in steps of 0.1 or 0.02° with Cu-Kα radiation (λ = 0.154 nm). Transmission Electron Microscopy (TEM) was used for observing the shape and the size of the LDHs using a JEM 2100F. The measurements were performed at room temperature. The different elements of the prepared materials were identified via energy-dispersive X-ray spectroscopy (EDX) using a JED 2300 Electron Probe Micro-Analyzer. Depending on molecular vibrational spectroscopic techniques, Fourier Transform Infrared Spectroscopy (FTIR) was used for determining the functional groups of the prepared materials using a Perkin Elmer Spectrum 400. Thermal analyses consisted of thermal gravimetric analysis (TGA) and differential scanning calorimetry (DSC). TGA, which was carried out using a TA thermogravimetric analyzer (series Q500), was used to characterize the decomposition and thermal stability of the materials under a nitrogen atmosphere. Using a TA series Q 600, DSC analysis was performed under the flow of inert gas with a heating rate of 10 °C min^−1^.

### 4.3. Adsorption Experiment

The removal efficiencies RE (%) of the modified structures, alongside GreensandPlus^TM^, were determined at 24 °C. An appropriate mass of the powder was stirred with a certain amount of freshly prepared multi-element standard solution containing some heavy metals of interest (Fe, Cu, Cr, Ni and Mn) for water filtration at pH 2~3 for 1 h to prepare aqueous solutions with constant concentrations W/V (2 g/L). After equilibrium (1 h) and using the centrifugation technique, the liquid part was extracted; then, the residual concentrations of heavy metals were measured using the ICPE technique (Inductively Coupled Plasma Emission Spectroscopy). By measuring the initial concentration (C_i_) and final concentration (C_f_) of the different metals (mg/L), the removal efficiency (RE%) could be determined through the following formula: RE (%) = (C_i_ − C_f_)/C_i_ × 100.

## 5. Conclusions

In the current study, nanolayered structures of Al/Zn were prepared and developed in three ways to produce stable and strong structures for working under severe conditions to purify water containing heavy metals. At the same time, increasing their performance was another target for the current study. In the first method, urea hydrolysis was used for preparing the standard nanolayered structure of Al/Zn LDHs, which showed high activity for removing iron. However, for the other metals (chromium, copper, nickel and manganese), lower activity was observed. The second method depended on the use of carbon nanotubes as fillers for the nanolayered structure. Although the stability of nanolayered structures increased, their activity for removing heavy metals decreased to low levels because CNTs, which combined with PVA, caused a blockage of the entrance gates to the interlayered region of the nanolayered structures. To avoid the blocking effect of CNTs, the third method was used to expand and widen the interlayered spacing of the nanolayered structures from 0.76 nm to more than 1.5 nm; this was achieved by building organic-CNT–PVA inorganic nanohybrids using long chains of hydrocarbons of stearic acid (CH_3_(CH_2_)_16_COOH) as pillars among the nanolayers of the nanolayered structures. In this way, the activity of the nanohybrid was very high for removing the different kinds of heavy metals. Compared with the conventional materials on the market, the current modified structures showed a couple of advantages, as shown in Table 3. The first one is the ability to work under severe conditions in a strong acidic medium. The second advantage is the ability to remove many of the heavy metal (Fe, Cu, Cr, Ni and Mn) ions in the same solution at the same time.

Finally, these experimental results concluded that the organic-CNT–PVA inorganic nanohybrids showed very effective results for removing the different kinds of heavy metals under severe conditions. In addition, achieving high activity of the prepared nanohybrids is a positive step in facing environment- and water-related problems brought about by rapidly growing industries. Furthermore, this study introduces promising new materials for purifying industrial water which has a strong acidic medium.

## Figures and Tables

**Table 1:** Applications of different types of nanomaterials and nanostructures.

Material Type	Application	References
Metal–organic nanohybrids	Removal agents of pollutants	[6]
Nanoparticles	Catalysts in industries	[7]
Gold nanohybrids	Drug deliveries	[8]
TiO_2_–SnO_2_ Nanocomposites	Gas sensing	[9]
Nanoparticles	Biological applications	[10]

**Table 2:** Original concentrations of some heavy metals in the analyzed mixed standard.

Heavy Metal	Conc. (mg/L)
Iron (Fe)	17.7
Copper (Cu)	18.7
Chromium (Cr)	18.2
Nickel (Ni)	14.4
Manganese (Mn)	20.2

**Table 3:** Summary of the removal percentages of heavy metals using different samples reported in the literature in comparison with the structures designed in the current study.

References	31	33	34	Commercial Material GSP	The Current Study
Operating Conditions	Under Normal Conditions			Under Severe Conditions	
Heavy Metals	Removal%				
Iron	-	88	-	62	76.6
Copper	100	55	31	0	50.2
Chromium	-	29	-	9	49.1
Nickel	-	-	-	0	45.15
Manganese	0.66	-	-	18	44.6

## Data Availability

The data are available in a publicly accessible repository.
